# Autoantibodies in Neuropsychiatric Systemic Lupus Erythematosus (NPSLE): Can They Be Used as Biomarkers for the Differential Diagnosis of This Disease?

**DOI:** 10.1007/s12016-021-08865-2

**Published:** 2021-06-11

**Authors:** Elias Manca

**Affiliations:** Department of Biomedical Sciences, University of Cagliari, 09042 Monserrato, Cagliari, Italy

**Keywords:** Autoantibodies, Neuropsychiatric Systemic Lupus Erythematosus, Biomarkers, Autoimmune diseases, Brain diseases

## Abstract

Systemic lupus erythematosus is a complex immunological disease where both environmental factors and genetic predisposition lead to the dysregulation of important immune mechanisms. Eventually, the combination of these factors leads to the production of self-reactive antibodies that can target any organ or tissue of the human body. Autoantibodies can form immune complexes responsible for both the organ damage and the most severe complications. Involvement of the central nervous system defines a subcategory of the disease, generally known with the denomination of neuropsychiatric systemic lupus erythematosus. Neuropsychiatric symptoms can range from relatively mild manifestations, such as headache, to more severe complications, such as psychosis. The evaluation of the presence of the autoantibodies in the serum of these patients is the most helpful diagnostic tool for the assessment of the disease. The scientific progresses achieved in the last decades helped researchers and physicians to discover some of autoepitopes targeted by the autoantibodies, although the majority of them have not been identified yet. Additionally, the central nervous system is full of epitopes that cannot be found elsewhere in the human body, for this reason, autoantibodies that selectively target these epitopes might be used for the differential diagnosis between patients with and without the neuropsychiatric symptoms. In this review, the most relevant data is reported with regard to mechanisms implicated in the production of autoantibodies and the most important autoantibodies found among patients with systemic lupus erythematosus with and without the neuropsychiatric manifestations.

## Introduction

Systemic lupus erythematosus (SLE) is a complex immunological disease where both environmental factors and genetic predisposition lead to the dysregulation of important immune mechanisms, such as cytokine production, apoptotic debris clearance, and aberrant immune cell activation. The hallmark of this disease is the production of self-reactive antibodies that may target any organ or tissue of the human body and eventually form immune complexes (ICs) which may accumulate in any organ of the body. The accumulation of ICs is responsible for the organ failure observed in patients affected by this disease [1]. SLE affects people worldwide, although each country exhibits its own incidence and prevalence. Moreover, the incidence and prevalence vary widely with sex, age, and ethnicity. Overall, both the incidence and prevalence of SLE are higher among women than men, with the former developing the disease earlier in life. Epidemiological studies showed that Afro-American people are at higher risk to develop this disease compared to Caucasian people, whereas the risk for Asian and Hispanic people is estimated to be in between [2]. The etiology of SLE is complicated and both environmental and hereditary factors contribute to the development of the clinical manifestations. Genetic variants alone cannot explain the clinical phenotype of patients affected by this disease. Twins studies showed that the concordance rate in monozygotic twins is about 25%, but only 2% in dizygotic twins [3]. Polymorphisms within the human leukocyte antigen (HLA) genes are associated with many autoimmune diseases. It is not surprising that these genes represent a well-known risk factor to patients with SLE polymorphisms. Moreover, HLA polymorphisms are also associated with increased risk of autoantibody production. Almost all patients with SLE at some point in life develop antibodies to self-antigens. HLA haplotypes consisting of DRB1*1501/DQB1*0602 (DR2) are associated with the presence of anti-Sm antibodies, while HLA DRB1*0301/DQB1*0201 (DR3) haplotypes are associated with anti-SSA/Ro and anti-SSB/La antibodies. Individuals with a mixture of DR2/DR3 haplotypes have an increased prevalence of anti-SSA/Ro, anti-SSB/La, and anti-Sm antibodies [4]. It was observed that among SLE patients with East-Asian ancestry, the production of antibodies such as anti-nuclear ribonucleoprotein (RNP) anti-SSA/Ro, anti-SSB/La, and anti-cardiolipins (aCL) is associated with variants on the HLA-DRB1 locus, while the production of anti-Sm antibodies is associated with variant on the HLA-DPB1 locus [5]. A common feature observed among patients with this disease consists in the aberrant activation of the immune response, leading to the loss of both the adaptive immune tolerance and to the dysregulation of the innate immune response. The fact that autoantibodies, in particular anti-nuclear antibodies (ANAs), can be detected years before the appearance of clinical symptoms has brought scientists to believe that the loss of tolerance to self is an important step in the development of SLE [6]. Anti-double-strand DNA (dsDNA), anti-SSA/Ro, anti-SSB/La, and anti-phospholipid antibodies can be found years before the clinical onset of the disease, whereas anti-Sm or anti-RNP antibodies can be found only few months before the appearance of the disease. The fact that many of these autoantibodies are found among individuals who will not develop this disease highly suggests for the presence of defects in the mechanisms controlling the peripheral expansion of autoreactive B cells [7, 8]. Additionally, the majority of pathogenic autoantibodies are both class-switched and somatically hypermutated immunoglobulin G (IgG), which are two phenomena that occur inside the germinal centers (GCs) [9]. In patients with SLE, B cell activation in ectopic GCs gives rise to autoreactive plasmablasts and plasma cells secreting high levels of autoantibodies.

## Mechanisms Leading to the Production of Autoantibodies

Several molecular mechanisms involved in the production of autoantibodies can be found altered among patients with SLE, regardless which tissue or organ is mostly affected. A remarkable number of patients with this disease experience an increase of the expression of interferon-associated genes compared to healthy individuals, a phenomenon known as *interferon signature*. The interferon signaling pathways play a crucial role in mechanisms of defense against viral infections, but they are also important for the activation and development of immune cells. During normal conditions, plasmatic levels of type 1 interferons (IFN-I) are low, but a peak can be seen soon after the establishment of a viral infection. Once the infection has been removed, the IFN-I levels decrease to the normal values. Viruses such as the Epstein Barr virus (EBV) can lead to chronic infections. IFN-I can stimulate the production of all subclasses of IgG and induce the differentiation of B cells into long-lived plasma cells and memory B cells [10]. Many patients with SLE have a documented previous infection with the EBV [11]. Epstein Barr virus nuclear antigen I (EBNA-1) was shown to trigger the production of anti-nuclear antibodies in patients affected by this disease [12]. Additionally, it was demonstrated that sustained IFN-I production, as observed during chronic viral infections, prevents the differentiation of activated CD4^+^ T cells into CD4^+^ T helper 1 (T_h1_) cells, but it does not prevent them to differentiate into CD4^+^ T follicular helper (T_FH_) cells. T_FH_ cells are essential for the germinal center formation and provide signals to B cells for their differentiation into plasmablasts and plasma cells that secrete high-affinity and isotype-switched antibody [13, 14]. The presence of anti-dsDNA and anti-SSA/Ro antibodies strongly correlates with high levels of interferon α (IFN-α) among patients with European, African, and Asian ancestral backgrounds [15]. There are studies reporting that also interferon γ (IFN-γ) levels are increased among patients with SLE compared with healthy subjects, although the exact role of IFN-γ in autoimmune syndromes is still controversial [16]. Nonetheless, experiments on animal models have showed the B cell–intrinsic deletion of the IFN-γ receptor (IFN-γR) abrogates autoimmune GC formation and class-switched autoantibody production [17]. The production of self-reactive antibodies requires both the presentation of self-antigens as non self and the failure of immune tolerance mechanisms. It is widely recognized that certain epitopes of pathogens, such as virus or bacteria that have an amino acid sequence similar to that of self-proteins can lead to the generation of self-reactive antibodies in several autoimmune disease. This phenomenon is known as molecular mimicry [18]. Neutrophil extracellular traps (NETs) are another mechanism believed to contribute to the failure of immune tolerance in SLE. NETs formation consists in the creation of a network of extracellular fibers containing nuclear antigens, such as DNA and histones, as well as pro-inflammatory proteins extruded by neutrophils. In patients affected by SLE, the defective removal of NETs is assumed to contribute to the overexposure of self-nuclear molecules to the adaptive and innate immune systems; this eventually induces the development of an immune response against nuclear self-antigens. The degradation of NETs is mediated by the action of the deoxyribonuclease I (DNase I), an enzyme that digests the DNA and histones forming the NETs. The enzymatic digestion of NETs leads to the formation of debris, that in normal conditions are immediately removed by macrophages [19]. It was proved that reduced capacity of DNase I leads to a longer exposure of NETs to immune cells, and this condition is thought to contribute to the production of anti-DNA antibodies [20]. The overexposure of self-antigens can also be attributable to the impaired clearance of apoptotic cells. On the surface of apoptotic cells is expressed phosphatidylserine (PS), which is recognized by phagocytes through the T cell immunoglobulin and mucin domain‐containing molecule 4 (Tim4). At the same time, PS can indirectly interact with phagocytes through several binding proteins, such as the milk fat globule EGF factor 8 (MFG‐E8) or the growth arrest–specific 6 (Gas6). The interaction between PS and these receptors induces the engulfment of apoptotic bodies by phagocytes [21, 22]. MFG‐E8 knockout mice show a decreased elimination of apoptotic cells due to a reduced phagocytic capacity of macrophages [21]. Miyanishi and colleagues showed that after 5 weeks of treatment with an anti-Tim-4 antibody, C57/Bl6 mice were characterized as having high serum levels of anti-dsDNA antibody [23]. It was shown that mice lacking the membrane tyrosine kinase c‐mer, a membrane protein used for bridging Gas6 to PS, are characterized by impaired clearance of apoptotic cells. These mice developed both lupus‐like autoimmunity symptoms and autoantibodies to chromatin and DNA [24].

Aberrant or defective interaction between B cells and other immune cells can lead to the hyper-proliferation of plasmablasts and plasma cells. Altered B cell subsets have been documented in patients with SLE and include elevated immature transitional B cells, memory B cells, plasmablasts, and circulating plasma cells [25–27]. Many factors control the B cell activation, differentiation, and function. Abnormal activation of B cells is a critical step in the initiation of SLE and can be induced either by aberrant cytokine release or by impaired protein membrane signaling, as shown in the Table 1. Common cytokines implicated in aberrant activation of B cells are BAFF, IL-6, IL-2, and IL-21, whereas membrane protein implicated in aberrant B cell signaling are CD40L/CD40, FcγRs, and TLRs. B cell activating factor (BAFF), also known as B lymphocyte stimulator or BLyS, is a member of the tumor necrosis factor superfamily and is produced mainly by myeloid cells and T lymphocytes. This cytokine is essential for B cell development and function. BAFF provides survival signals and promotes class switch recombination by binding its receptor (BAFF-R) on B cells [28, 29]. Elevated levels of BAFF inhibit B cell receptor (BCR)-induced cell death by promoting the downregulation of the proapoptotic protein Bim [30]. Experiments on transgenic mice overexpressing BAFF displayed a lupus-like phenotype with increased B cells, hypergammaglobulinemia, high-titer serum anti-DNA antibody, and IC deposition in the kidneys [29]. In patients with SLE, serum BAFF levels positively correlate with the anti-dsDNA antibody titer, although changes in serum levels of this cytokine do not correlate with changes in the disease activity [31, 32]. Interleukin 6 (IL-6) is a potent proinflammatory cytokine. Among patients with SLE, levels of this cytokine can be found increased at the same time or just before the appearance of the autoantibodies during preclinical SLE. This suggests for a critical role of this cytokine in the dysregulation of B and T cell tolerance among patients with this disease [33]. B cells isolated from patients with SLE produce high level of IL-6. A study on mice showed that the Il-6 produced by B cells can induce the differentiation of T_FH_ cells and lead to the spontaneous formation of germinal centers [34]. Another characteristic feature of patients with SLE is the decreased production of interleukin-2 (IL-2), which is an important cytokine for the maintenance of CD4^+^ T regulatory (T_reg_) cells [35]. It was shown that among patients with SLE, the number of T_reg_ cells is lower compared with healthy subjects. In contrast, the number of other T cells subpopulations with a pro-inflammatory phenotype, such as CD4^+^ T helper 17 (T_h17_) and memory CD8^+^ cytotoxic cells, is usually increased, and this condition associates with a poor prognosis of SLE [36, 37]. Interleukin 21 (IL-21) is mainly secreted by T_FH_ and T_h17_ cells, while its receptor is found on both naïve and activated B cells located within the GCs. The role of this cytokine is to amplify the proliferation mediated by CD40/CD40L interaction [38]. In addition, acting in combination with CD40, this cytokine mediates the proliferation, differentiation, and class switch recombination in plasmablasts and plasma cells [39]. High levels of IL-21 are found in patients with SLE. It was demonstrated that B cells isolated from SLE patients and treated with a monoclonal antibody against IL-21 receptor cease to proliferate [40, 41]. CD40 ligand (CD40L), a member of the tumor necrosis factor superfamily, is expressed on naïve and activated CD4^+^ T cells, whereas its receptor CD40 is expressed on B cells and antigen-presenting cells (APCs). CD40/CD40L interaction promotes GC formation and leads to B cell differentiation, isotype switching, and the formation of long-lived plasma cells and memory B cells [42]. In patients with SLE CD40L is over-expressed on both CD4^+^ T cells and CD8^+^ T cells. Ectopic expression of CD40L on B cells was observed both in SLE patients and in lupus-prone mice. In addition, the soluble form of CD40L levels was correlated with the autoantibody titers and disease activity in SLE [43, 44]. Whereas CD40 engagement on the surface of dendritic cells (DCs) induces their cytokine production and the expression of costimulatory molecules on their surface, facilitating the cross-presentation of antigens to the adaptive immune system [42]. Fcγ receptors (FcγRs) are transmembrane proteins capable of binding the Fc region of IgG and belong to the immunoglobulin superfamily. Several members of this receptor family exist, and many of them can be found expressed on B cells and on DCs [45]. The interaction between ICs and FcγRs triggers intracellular signaling pathways that play a pivotal role in the autoimmune response. The FcγRIIB is an inhibitory Fc receptor which can interact with both the BCR and ICs. Signaling though FcγRIIB induces the phosphorylation of the cytoplasmic domain of the immunoreceptor tyrosine-based inhibitory motif (ITIM) by the Src-family kinase LYN. LYN plays an essential role in the transmission of inhibitory signals that attenuate B cell activation, thus mediating the immune tolerance [46]. It was observed that lupus-prone mice express low levels of FcγRIIB. Additionally, it was shown that increased expression of FcγRIIB on B cells can reduce both anti-dsDNA antibody levels and proteinuria in lupus-prone mice [47]. B cells can also be activated by Toll-like receptors (TLRs), such as TLR9 and TLR7. TLR9 is constitutively expressed on human B cells, whereas the expression of TLR7 on B cells is induced by IFN-I [48]. TLR7 and TLR9 recognize single-stranded RNA and DNA, respectively. B cells with DNA-reactive BCR can be activated by DNA molecules synergistically via the BCR and TLR9 [49–51]. In lupus-prone mice, TLR9 mediates production of anti-dsDNA and anti-chromatin autoantibodies, and the increased expression of TLR9 associates with higher disease activity in SLE patients [52]. Although TLR7 deletion ameliorated disease in MRL/lpr mice, it does not prevent the production of autoantibodies against RNA-containing antigens [53]. In addition, TLR7 but not TLR9 can induce the IFN-α gene expression [54].

**Table 1:** Summary of the main cytokines and membrane receptors involved in the production of autoantibodies among patients with SLE

Name	Type of molecule	Change	Function
IFN-I	Cytokine	Increased	– Promotes the differentiation of long-lived plasma cells and memory B cells – Stimulate the production of all subclasses of IgG – Induces the differentiation of T_FH_ cells
BAFF	Cytokine	Increased	– Promotes the survival of B cells – Promotes the downregulation of Bim – Inhibits BCR-mediated cell death
IL-6	Cytokine	Increased	– Promotes spontaneous GCs formation – Induces the differentiation of T_FH_ cells
IL-2	Cytokine	Decreased	– Promotes the differentiation of T_reg_ cells
IL-21	Cytokine	Increased	– Promotes the proliferation of B cells mediated by CD40/CD40L interaction
CD40/CD40L	Membrane receptor	Increased	– Promotes GC formation – Promotes the differentiation of B cells – Promotes the antibodies isotype switching
FcyRIIB	Membrane receptor	Decreased	– Inhibits the activation of B cells
TLR-7/9	Membrane receptor	Increased	– Promotes the activation and differentiation of B cells – Promotes autoantibodies productions

## Neuropsychiatric Systemic Lupus Erythematosus

Neuropsychiatric systemic lupus erythematosus (NPSLE) is one of the poorest understood and complex subcategories of SLE. Patients affected by NPSLE can show a wide variety of symptoms, ranging from mild manifestations, such as headache, to more severe complications, such as cognitive dysfunctions. It is estimated that between 17 and 75% of patients with SLE develop neuropsychiatric symptoms during their disease course [55]. Neuropsychiatric manifestations can affect both the central nervous system (CNS) and the peripheral nervous system (PNS); besides, symptomatology of the CNS can be divided into focal and diffuse manifestations as shown in Table 2 [56, 57]. Diffuse symptoms seem to be related to the inflammation elicited by different mediators, such as IFN-α, anti-*N*-methyl-d-aspartate (NMDA) receptor antibodies and anti-ribosomal P protein antibodies (anti-ribP), which have been associated with the leakage of the blood–brain barrier (BBB) [58, 59]. While focal manifestations have been associated with the presence of autoantibodies targeting the membrane phospholipids of endothelial cells of blood vessels within the CNS; additionally, their presence in the CSF correlates with events such as cerebral vasculopathy, thrombosis, and complement activation [60].

**Table 2:** Classification of the manifestations involving the CNS and the PNS among patients with NPSLE

Central nervous system		Peripheral nervous system
Focal	Diffuse	
– Seizure disorders	– Headache	– Guillain–Barre' syndrome
– Cerebrovascular disease	– Myelopathy	– Automatic disorder
– Demyelinating syndrome	– Cognitive dysfunction	– Mononeuropathy, single/multiplex
– Movement disorder	– Mood disorder	– Neuropathy, cranial
– Aseptic meningitis	– Psychosis	– Myasthenia gravis
– Anxiety disorder	– Acute confusional state	– Plexopathy
	– Myasthenia Gravis disorder	– Polyneuropathy

**Fig. 1:**
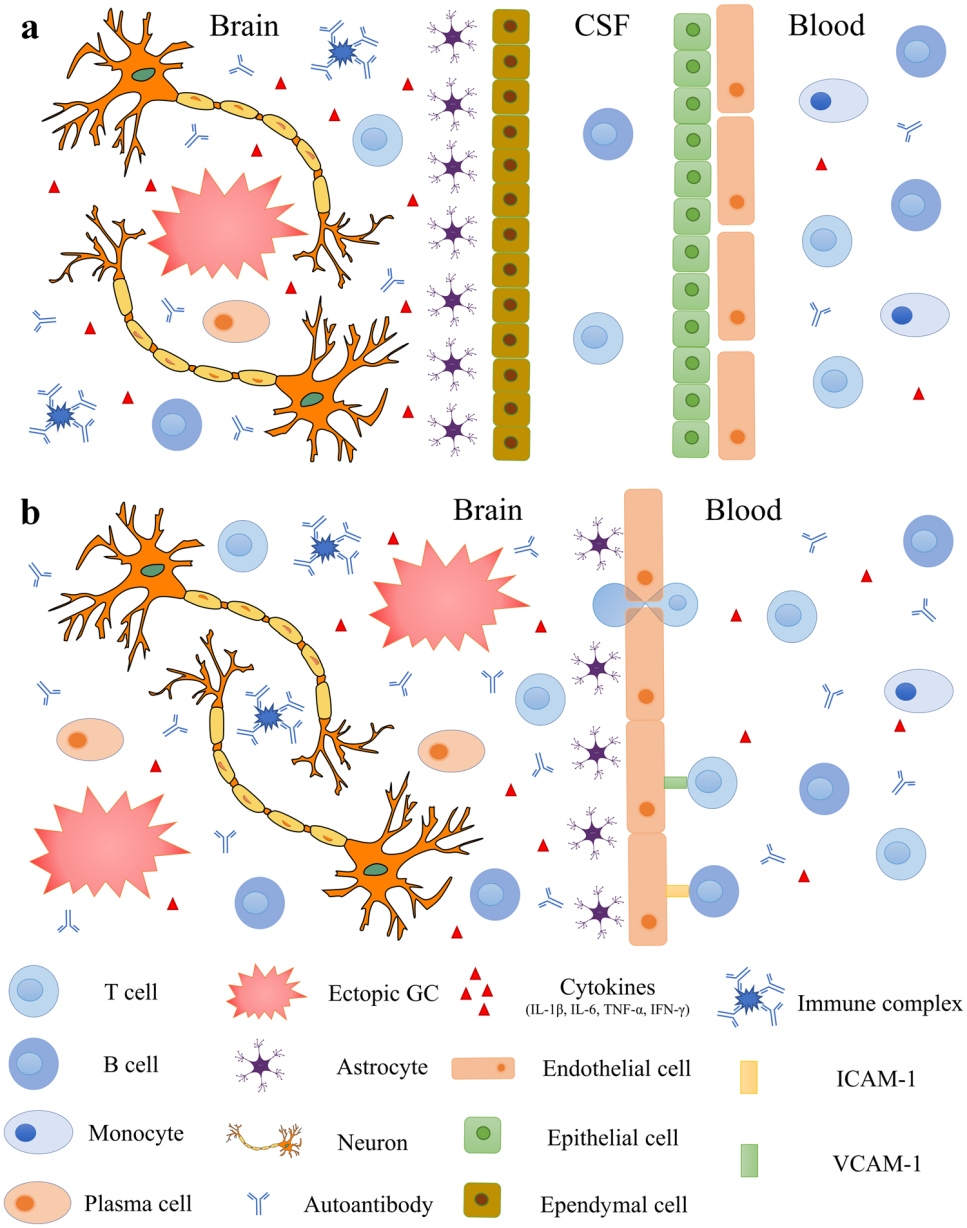
Schematic representation of how autoantibodies can enter the brain parenchyma. **a** During normal conditions, leucocytes can enter the brain parenchyma passing through the choroid plexus in the brain ventricles. Malfunctioning leucocytes can be activated and start an immune response which lead to the formation of ectopic germinal centers and the production of autoantibodies. Eventually, autoantibodies associate to generate immune complexes which accumulate in the brain and damage the neurons. **b** High levels of cytokines, such as IL-1β, IL-6, TNF-α, and IFN-γ, can induce the expression of ICAM-1 and PECAM-1 molecules on the surface of endothelial cells and facilitate the extravasation of leukocytes across the blood brain barrier. Inside the brain, the malfunctioning leukocytes can be activated and initiate an immune response and lead to the generation of ectopic germinal centers and the production of autoantibodies. Eventually, autoantibodies associate to generate immune complexes which accumulate in the brain and damage the neurons

The etiology of NPSLE can be due to different mechanisms, such as autoantibody production, microangiopathy, BBB disruption, intrathecal production of proinflammatory cytokines, and premature atherosclerosis. Cohen and colleagues performed a post-mortem study of the brain histopathology from patients with NPSLE. In their study, it was shown that morphological alterations, such as microinfarctions, macroinfarctions, diffuse vasculopathies, and microthrombi, can happen both in patients with and without neuropsychiatric manifestation. However, these lesions were significantly more often observed among patients with neurological manifestations. Focal vasculopathies were observed both in patients and healthy controls, the reason why diffuse manifestations appear to play a pivotal role in the development of neurological manifestations among patients with SLE. Moreover, their study demonstrated for the first time that deposits in the brain of the classical complement components C1q and C4d, and the terminal complement complex (C5b-9), are associated with the development of brain thrombosis and ischemia [61]. Studies on lupus prone mice showed that one of the earliest brain morphological changes associated with neurological symptoms is the atrophy of the pyramidal neurons of the parietal cortex and CA1 region of the hippocampus [62]. Focal symptoms of NPSLE are believed to be caused by thrombotic events or strokes, and these events are always followed by the leakage of BBB. For a long time, the leakage of the BBB was believed to be the only mechanism allowing peripheral autoantibodies to enter the brain parenchyma and start an immune response to neuronal components. Goral and colleagues studying patients affected by NPSLE have discovered that the presence of aCL, commonly associated with thrombosis risk in patients with SLE [63], is not associated with BBB disruption or intrathecal antibody production [64]. These results suggest that the disruption of BBB is not due to prothrombotic events, but rather it is related to the overexpression of proinflammatory cytokines promoting the infiltration of leukocytes within the CNS (Fig. 1). High levels of interleukin 1β (IL-1β), IFN-γ and IL-6 have been observed in the serum of patients affected by SLE [65]. IL-1β is a potent inducer of intercellular adhesion molecule 1 (ICAM-1) and vascular cell adhesion protein 1 (VCAM-1) on the surface of endothelial cells, thus promoting the adhesion and the consequent migration of T cells across the BBB [66]. Experiments on mice proved that high levels of IL-1β promote the migration of CCR2^hi^Ly6C^hi^ monocytes through the blood-spinal cord barrier (BSCB) and their acquisition of APCs phenotype, followed by the activation of autoreactive CD4^+^ T cells [67]. In patients affected by SLE, high levels of IFN-γ promote the entrance of autoreactive CD4^+^ T cells in the CNS, by acting on endothelial cells and inducing them to localize on their surface molecules such as ICAM-1, platelet endothelial cell adhesion molecule (PECAM-1). The overexpression of ICAM-1 and VCAM-1 on the surface of endothelial cells promotes also the migration of B cells within the CNS [68]. Patients with SLE are characterized by high plasmatic levels of IL-6, and this cytokine is known to promote the activation of both B and T cells outside germinal centers [69]. Although high levels of IL-6 are mostly produced peripherally, this cytokine can also be synthetized within the CNS by astrocytes [70, 71]. These events may lead to the formation of ectopic germinal center within the brain as well as the production of self-reactive antibodies. Gelb and colleagues investigated the source of intrathecal antibodies in NPSLE using MRL/lpr mice, a spontaneous mice model for SLE where mice develop neuropsychiatric symptoms. They observed the accumulation of IgG in the ependymal layer and in brain parenchyma of sub ventricular zones at the lateral, third, and fourth brain ventricle. However, none of their mice displayed changes in the cell structure of the endothelial cells in the BBB. Taking these findings together, the only way immunoglobulins can reach the brain parenchyma is across the blood-cerebrospinal fluid barrier (BCSFB). Hence, antibodies extravasate from the fenestrated capillaries of the choroid plexus into the choroid plexus stroma, and from here, antibodies might abnormally cross the dysfunctional BCSFB to enter the CSF, and then accumulate in the proximity of the brain ventricles [59]. It is well known that cytokines not only regulate the immune response, but they can also modulate neuronal functions. Changes in the serum levels of IL-6 and TNF-α are associated with depression-like behavior both in human and in mice model [72, 73]. Studies on the MRL/lpr mice model demonstrated that high levels of IL-6 are associated with anhedonia, and treatment with cyclophosphamide, a chemotherapeutical used to suppress the immune system, can repress this behavior [74]. In the CSF from patients affected by NPSLE can be found high levels of cytokines such as IL-1, IL-6, TNF-α, and IFN-α [75]. Cytokines such as IL-1β, IL-6, and IFN-γ are found at high levels in patient serum [76]. High levels of tumor necrosis factor alpha (TNF-α) in patients with NPSLE are associated with diffuse symptoms [77]. TNF-α can be synthetized by both neurons and astrocytes [78, 79]; however, the main source of this cytokine in the CNS comes from microglia cells [80]. Low levels of TNF-α modulate the synaptic plasticity of hippocampal cells, by controlling the trafficking of AMPA-type glutamate receptors (AMPARs) on these cells. [80]. TNF-α increases the expression of glutamate receptor 1 (GluR1)-containing AMPARs, which is required for the preservation of the synaptic strength [81]. High levels of this cytokine induce the apoptosis of hippocampal cells, by signaling through the tumor necrosis factor receptor 1 (TNFR1) [79]. During physiological conditions, the CNS does not produce enough amount of TNF-α to induce the apoptosis of hippocampal neurons; however, patients affected by NPSLE are characterized by both BBB dysfunction and high circulating levels of IFN-γ [59, 76], which is a potent inducer of TNF-α [82]. Activation of the TNFR1 on hippocampal neurons is associated with preferential increase of glutamate receptor 2 (GluR2)-lacking AMPARs. GluR2 is more permeable to Ca^2+^ than other ions, and high levels of cytoplasmatic calcium can initiate the death by apoptosis of these neurons [83]. TNF-α plays a role in controlling the trafficking of NMDA receptors on hippocampal neuron cultures, thus modulating their synaptic plasticity [84]. TNF-α is also able to potentiate NMDA receptor–mediated excitotoxicity in cortical neurons [85]. Increased levels of IL-6 and TNF-α have been associated with different brain disorders, such as schizophrenia and major depressive disorder [86, 87]. Increased mRNA expression of IL-6, IL-1β, and TNF-α in the hippocampus of db/db mice have been associated with cognitive impairment [88]. Central administration of recombinant mouse IL-6 produced depressive-like phenotypes in Swiss Webster mice; this is further confirmed by the fact that IL-6 knockout mice show a reduced depression-like behavior, compared with wild-type mice [89, 90].

## Systemic and Brain-Targeting Autoantibodies in SLE and NPSLE

### Autoantibodies to Systemic Components

The production of autoantibodies is a hallmark of SLE, and their production is directly linked to the tissue damage and organ failure observed in patients with this disease [91]. The human adaptive immune system is able to produce antibodies to a nearly unrestricted number of antigens; therefore, every patient with this disease displays a unique signature of (auto)antibodies. Nonetheless, there are specific autoantibodies that recurrently found the serum and CSF of patients with this disease. The main autoantibodies found among patients with NPSLE are listed in the Table 3. Autoantibodies that specifically bind antigens inside the nucleus of the cells are the distinctive characteristic of SLE, although cytoplasmatic components can be also be targeted as well. Antinuclear antibodies (ANAs) are a helpful biomarker for the detection of many autoimmune disorders, such as SLE and Sjogren’s syndrome (SS). The ANAs are a heterogenic group of antibodies that recognize self-antigens within the nucleus of cells, such as small nuclear ribonucleoproteins (snRNPs), histone proteins, dsDNA, DNA/histone complexes, various nuclear enzymes, and other proteins. It is well established that high ANAs are found healthy subjects, in a range between 20 and 30%; however, in autoimmune conditions, their titer is often found increased [7]. High levels of ANAs are often observed among patients with NPSLE. The role of ANAs for the diagnosis of NPSLE was evaluated by several studies. Unfortunately, the detection of ANAs among patients during their first psychiatric episode was revealed to be unspecific for the diagnosis of this disease, mostly because of the false positive results among patients under certain pharmacological treatment [92, 93]. Extractable nuclear antigen (ENA) antibodies are a subgroup of ANAs so called because they are acid non-histone macromolecules obtained from the saline soluble fraction of cell nuclei. Anti-ENA antibodies are a heterogenous group of antibodies that can target many different antigens, such as anti-Sm, anti-RNP, anti-SSA/Ro, and anti-SSB/La. Anti-ENA antibodies are considered to be more sensitive than ANAs; indeed, anti-RNP antibodies can be detected at the dilution 1:10^6^, whereas ANAs in the same sample are detected only at the dilution of 1:40. In addition, some anti-ENA antibodies can be detected outside the nucleus, making the term *nuclear antigen* a kind of misnomer. Around 50 to 60% of patients with NPSLE are positive for these antibodies [94, 95]. Since many patients with NPSLE are positive to anti-ENA antibodies, some researchers and physicians believe that these autoantibodies play a role in the immune dysregulation of this disease [96]. Anti-Sm antibodies are directed against the protein core of snRNPs, while anti-RNP antibodies target the protein A and the protein C of the U1 snRNPs. Anti-Sm antibodies are detected among SLE patients in a percentage between 5 and 30% while anti-RNPs a detected in 25 and 47% of SLE patients. Whereas among patients with NPSLE anti-Sm and anti-RNP are detected in a range between 18 and 48% and 18 and 60%, respectively [97]. The presence of Anti-Sm antibodies is associated with the breakdown of the BBB and acute confusional states, among patients with NPSLE [98, 99]. These two types of antibodies are almost always found together in patients with SLE, and in those few cases in which anti-Sm alone was initially detected, anti-RNP developed later during the course of the disease [100]. Anti-SSA/Ro and anti-SSB/La antibodies recognize respectively the protein Ro and La. Ro and La proteins associate together and bind to Y RNA, which are small non-coding RNAs involved in the chromosomal DNA replication [101, 102]. Anti-SSA/Ro antibodies are widely found among many autoimmune diseases, such as SLE and SS. In contrast, anti-SSB/La are typically found only in patients affected by SS, and if they are detected among patients with other autoimmune disorders, anti-SSB/La antibodies are always accompanied by anti-SSA/Ro antibodies [103]. Some studies associate the presence of anti-SSA/Ro, but not anti-SSB/La, antibodies with the development of NPSLE [97]. It was showed that anti-SSA/Ro and anti-SSB/La antibodies are found between 36 and 64% and 8 and 34% of patients with SLE, respectively [104]. Whereas among patients with NPSLE, anti-SSA/Ro and anti-SSB/La antibodies can be observed in a range between 36 and 86% and 14 and 32%, respectively [97]. Autoantibodies to histones target the five major classes of histone proteins: H1, H2a, H2b, H3, and H4. They can be found in around 60% of patients with NPSLE [105]. Although they do not associate with any neurological manifestation, these autoantibodies correlate with the activity of the disease. However, in some studies, it is reported that the presence of autoantibodies that specifically bind to H1 and H3 fractions correlates with the presence of neuropsychiatric symptoms [106, 107]. Antibodies that specifically bind to dsDNA molecules are those that carry the most relevant diagnostic power and clinical application. These autoantibodies can be both IgM or IgG class; however, IgG class antibodies have a higher clinical relevance because they are more likely to include high-affinity subgroups with narrow cross reactivity [108]. It was observed that high levels of arginine, lysine, and asparagine residues in complementarity-determining regions of IgG anti-dsDNA antibodies facilitate the binding with the negatively charged DNA molecules. A recent study confirmed that anti‐dsDNA antibodies of patients with SLE have a specific immunoglobulin variable region signature; thus, the proteomic sequencing of these regions could be a valid tool for the diagnosis and clinical follow-up of patients with SLE [109, 110]. The number of patients with SLE positive for of these antibodies ranges between 60 and 90%, while among patients with NPSLE, they can be observed in 81% of patients [111]. Anti-dsDNA antibodies’ presence in patient’s serum fluctuates with the course of the disease, and they are usually detected in more than 60% of patients with active disease, while they are regularly found in only 10–15% of patients with inactive disease [112, 113]. Furthermore, anti-dsDNA antibodies can be detected several years before the clinical onset of the disease [6]. Interestingly, among patients with NPSLE, the presence of anti-dsDNA associates with poor performance of visuospatial skills, attention, and executive function [114]. The term Lupus anticoagulant (LAC) indicates a heterogenous class of immunoglobulins that specifically target the epitopes of the negatively charged phospholipid binding proteins, cardiolipins, prothrombin, and β2-glycoprotein I. These antibodies are not specific for SLE, since they are found in other disorders of the immune system with the impairment of the coagulation process [115]. The presence of LAC is strongly associated with stroke, transient ischemic attack, transverse myelitis, epilepsy, chorea, and dementia [116–119]. These autoantibodies can be found in one quarter of patients with SLE [120]. Among patients with NPSLE, aCL antibodies are commonly found in a range between 10 and 14.5% and are associated with development of headache, acute psychosis, cognitive impairment, high cortical dysfunction, and altered consciousness [97, 121, 122]. High levels of aCL antibodies can be found both in the serum and CSF of patients affected by NPSLE. To understand whether aCL antibodies were generated in the CNS or they were entering in the CSF from the systemic circulation, Cordero and colleagues measured the Q-albumin index of patients with NPSLE with high levels of aCL antibodies. It was observed that in some patients, the Q-albumin index was abnormal, which is suggestive for the disruption of the BBB; therefore, the aCL antibodies were generated outside the CNS. However, in other patients, this index was normal, which suggested for the intrathecal production of these autoantibodies [123]. This data suggests that both mechanisms can be responsible to the development of neuropsychiatric symptoms in patients affected by SLE. Autoantibodies that recognize the C-terminal sequence shared between the three acid ribosomal phosphoproteins P0, P1, and P2 can be found among patients with SLE [124]. Anti-ribP antibodies can be found at high frequency among patients with SLE. Among patients with NPSLE, anti-ribP antibodies can be found in a range from 10 to 47% and strongly correlate with neuropsychiatric manifestation [125–127]. Interestingly, Bonfa and colleagues have observed that 90% of SLE patients with psychosis possess these antibodies [128]. Anti-ribP antibodies strongly associate with neuropsychiatric, skin manifestations and the juvenile onset of SLE [129–131]. These autoantibodies have been found also in the CFS of patients affected by NPSLE, and the levels of these antibodies were significantly higher among patients with diffuse symptoms rather than patients with focal symptoms [132]. In another study, it was observed an association between the presence of anti-ribP and anti-dsDNA antibodies in patients affected by lupus nephritis [133]. Sun and colleagues have shown that anti-ribP antibodies concentration fluctuates according with disease activity [134]. Other studies associate the presence of anti-ribP antibodies with psychosis and depression; however, it must be reported that there are other studies refuting this association [135–137]. Nevertheless, to date, the anti-ribP antibodies are considered as the best biomarkers for the diagnosis of NPSLE [138].

**Table 3:** Autoantibodies observed among NPSLE patients

Antibody	Target	Frequency	Localization	Correlation with the neurologic manifestations
ANAs	Nuclear antigens	50–60%	Systemic	Their increase correlates with the activity of the disease
Anti-dsDNA	Double stranded DNA	~ 80%	Systemic	Their increase correlates with the activity of the disease
Anti-Histone	Histones	~ 60%	Systemic	Their increase correlates with the activity of the disease
Anti-Sm	Protein core of snRNPs	18–48%	Systemic	High levels correlate with acute confusional state and disruption of the BBB
Anti-RNP	Protein core of U1 snRNPs	18–60%	Systemic	Not a clear correlation between these autoantibodies and neuropsychiatric manifestations
Anti-SSA/Ro	Ro antigen	36–86%	Systemic	Their presence associates with NPSLE
Anti-SSB/LA	La antigen	14–32%	Systemic	Their presence does not associate with NPSLE
Anti-ribP	Acid ribomal phosphoproteins	10–47%	Systemic	High levels correlate with psychosis
aCL	Cardiolipins	10–14.5%	Systemic	Their presence associates with headache, acute psychosis, cognitive impairment, high cortical dysfunction, and altered consciousness
Anti-NMAD receptors	N-methyl-D-aspartate receptor	~ 60%	Brain specific	Their presence associate with CNS manifestations, specifically with diffuse symptoms such as cognitive dysfunction
AGA	Gangliosides	15.5–29.40%	Brain specific	Their presence associates with migraine, dementia, and peripheral neuropathy
Anti-MAP-2	Map-2	17%	Brain specific	Their presence associates with neuropsychiatric symptoms
ANFA	Neurofilaments	41%	Brain specific	Their presence is observed among patients with diffuse symptoms
Anti-TPI	Triose phosphate isomerase	42.90%	Brain specific	Their presence associates with psychosis, seizures, demyelinating syndrome, depression, and polyneuropathy

### Autoantibodies to Brain Components

 Brain-targeting autoantibodies have been found both in the serum [139] and in the CSF [140] of patients with NPSLE. Some of the brain-specific antibodies recognize a specific antigen, such as anti-NMDA receptors, whereas the target of other brain-targeting antibodies has not been identified yet. These autoantibodies are found in around 25 to 33% of patients with SLE and around 60% in patients with NPSLE [141–143]. The NMDA receptors belong to the family of ionotropic glutamate receptors, and they can be composed of three different subunits, named, NR1, NR2, and NR3. Gaynor and colleagues showed the ability of R4A, a mouse monoclonal IgG2b anti-dsDNA, to bind the amino acid sequence DWEYS. Later, it was demonstrated that this amino acid sequence can elicit both the immune response and anti-dsDNA antibodies production in BALB/c mice [144, 145]. This sequence is also found in the extracellular domain of human NR2 subunits. In addition, autoantibodies that cross-react with DNA and NMDA receptors have been found in the cerebrospinal fluid of SLE patients and are believed to mediate non-thrombotic and non-vasculitic abnormalities of the central nervous system [146]. It was demonstrated that anti-NMDA receptor antibodies target the hippocampus of mice and lead to the impairment of both spatial cognition and spatial memory through aberrant excitatory signaling, apoptosis, dendritic pruning, and microglial activation [147, 148]. Anti-NMDA receptor antibodies are prevalently found among patients with NPSLE suffering from CNS symptoms, and only in few cases are observed among patients showing PNS symptoms [111]. In addition, the presence of these autoantibodies among patients with NPSLE associates with the development of cognitive dysfunction [149]. Although the autoantibodies to NMDA receptors are the most studied brain-targeting antibodies in patients with SLE, there are other studies reporting the presence of self-antibodies to other brain molecules. Antibodies against gangliosides (AGAs), in a range between 15.5 and 29.4%, were found in patients with NPSLE [150, 151]. One study showed a strong correlation between IgG AGA in the CSF and IgM AGA in the serum and the presence of neuropsychiatric manifestations among patients with SLE [152]. Another study associated the presence of AGA IgG with symptoms such as migraine, dementia, and peripheral neuropathy; additionally, it was observed that the presence of AGA associates with HLA-DQB1*0605 haplotype [151]. AGA was also found in the CSF of 9 children with NPSLE. No ANAs were found in the CSF of these children, although they possessed a high ANA titer in their serum [153]. This indicates that the BBB was intact, and that the autoantibodies were presumably intrathecally produced. However, it should be reported that Martinez and colleagues in their study failed to find a clear correlation between the presence of AGA and neuropsychiatric symptoms in patients with SLE [150]. More studies should be carried on to better clarify the presence of AGA in patients affected by NPSLE. Williams and colleagues reported the presence of self-reactive antibodies to microtubule associated protein 2 (MAP-2) in 17% of patients affected by SLE, and their presence strongly correlates with neuropsychiatric symptoms. However, they also found a significant presence of these autoantibodies in control patients affected by other neurologic diseases or patients with brain injuries, therefore reducing the specificity of anti-MAP-2 antibodies as biomarker for NPSLE [154]. Immunoblotting analysis identified in the sera and CSF of patients with NPSLE the presence of autoantibodies targeting neurofilaments (ANFA). In the serum of patients enrolled for the study were found autoantibodies recognizing mostly the medium molecular weight size (160 kDa) and the high molecular weight size (205 kDa) of neurofilaments, whereas only one patient showed to have antibodies that recognized low molecular weight size neurofilament (70 kDa). Overall, 41% of patients enrolled in this study showed to have developed ANFA. Interestingly, in this study, the presence of ANFA was detected mainly in patients with diffuse symptoms [155]. There is only one study reporting that autoantibodies from the sera of patients affected by the NPSLE react specifically to glycoproteins present in the synaptosome isolated from Sprague–Dawley rats; however, it was not specified which exact synaptosomal glycoproteins were recognized by the autoantibodies [156]. Another study identified both in the sera (42.9% of patients with NPSLE) and CSF from patients affected by NPSLE self-reactive antibodies to triose phosphate isomerase (TPI), and these antibodies were associated with psychosis. The most common neuropsychiatric manifestation was psychosis, but other symptoms observed were seizures, demyelinating syndrome, depression, and polyneuropathy [157]. Patients were positive for anti-TPI antibodies where TPI plays an important role in the glycolysis, and deficiencies of this enzyme lead to a severe neurologic syndrome called triose phosphate isomerase deficiency [157, 158]. In one study it was showed that in patients with NPSLE is present a subset of autoantibodies that bind to Guanosine diphosphate dissociation inhibitor α (αGDI). However, experiment of immunocytochemistry on SH-SY5Y cells showed a minimal colocalization between the anti-αGDI monoclonal antibody and the autoantibodies present in the serum of patients with NPSLE [167]. Other studies tried to identify autoantibodies directed to neuronal components; however, in these studies, a specific target could not be identified. Huston and colleagues identified autoantibodies that reacted with a 50-kDa antigen in the plasma membrane of synaptic terminals. These antibodies were found in the plasma of 95% of patients with NPSLE and in the 35% of patients with SLE without the neuropsychiatric manifestations, while these autoantibodies were not found either in healthy subjects or in case control patients (except for the case of a patient with rheumatoid vasculitis suffering from organic brain syndrome). The presence of autoantibodies recognizing a protein of 50 kDa was also observed in the CSF of the same patients [159]. Another study described the presence of brain reactive antibodies in the serum of patients affected by SLE and with neuronal manifestation. Immunoblotting analysis demonstrated that these antibodies react with protein with a molecular weight between 27.5 and 29.5 kDa. Moreover, the presence of these antibodies highly associated with seizures and psychosis [160].

## Conclusions

SLE is a complex disease, and patients with this disease show different manifestations, reasons why its diagnosis is not easy or immediate. Currently, the diagnosis of SLE is based on the anamnestic observation and on a few blood biomarkers. The levels of these biomarkers can be found altered among patients suffering from other diseases. In the case of NPSLE, the diagnosis is even harder. Patients suffering from this disease can be affected by several different neurological manifestations, ranging from stroke to psychosis. These neurological manifestations are observed among patients suffering from other brain diseases or brain injuries. All these factors make the NPSLE one of the most difficult subcategories of SLE to be identified. Even if there is not a cure for patients with this disease, a prompt treatment is helpful to prevent a long-term morbidity and to decrease the mortality risk [91]. Finding a biomarker which is specific and sensible for the NPSLE would help physicians in the prompt diagnosis of this disease. Such biomarker should be present only in patients with NPSLE and permit the detection of the disease at the first neuropsychiatric episode. Moreover, such biomarker should be easy to test. Reason why the detection of this biomarker should be achieved through a blood sample, rather than through other techniques (such as the magnetic resonance imaging) which might be not only annoying or uncomfortable for the patient, but also time consuming for a rapid diagnosis. Good candidate biomarkers for the detection of NPSLE are the autoantibodies. Autoantibodies are currently the most powerful tool for the diagnosis of NPSLE. However, not all of them are characterized by high specificity and sensitivity for the NPSLE. The presence of ANAs or anti-dsDNA antibodies can be found in every patient with SLE and not only in those with NPSLE. The frequency of other antibodies, such as anti-SSA/Ro, anti-SSB/La, anti-Sm or anti-RNP, found among patients with NPSLE varies widely among the different cohorts that have been studied. LAC is a group of autoantibodies frequently found among patients with NPSLE, and usually associated with focal symptoms; however, these autoantibodies can be found in other diseases, such as Behcet’s disease or syphilis [161]. The gold standard for the differential diagnosis of NPSLE is to use a biomarker restricted in the brain and present only among patients with neuropsychiatric manifestations. The NMDA receptor is an autoantigen that is widely distributed in the brain, and its expression outside the CNS is restricted to few organs [162]. Even if anti-NMDA receptor antibodies are more frequently found in patients with neuropsychiatric manifestations than in patients without neurological involvement, their presence is not equally distributed between patients with diffuse and focal symptomatology. The presence of these autoantibodies among NPSLE patients associates prevalently with diffuse symptoms, such as depression and cognitive dysfunctions [142, 163]. Despite these studies reporting a correlation between anti-NMDA receptor antibodies and NPSLE, there are other studies that failed in demonstrating this correlation [164–166]. Although anti-ribP antibodies are not targeting a brain-restricted antigen, they seem to be a promising biomarker for the diagnosis of NPSLE, able to predict the flare of the disease along with anti-dsDNA antibodies. Because of these reasons, it is now considered the most reliable biomarker for the diagnosis of NPSLE. However, their presence associates only with diffuse symptoms, and it must be reported that there are studies showing opposite results concerning their role as biomarker for the diagnosis of NPSLE. There is no exclusive biomarker able to predict the development of neuropsychiatric manifestation among patients with SLE. However, several autoantibodies can be currently tested in order to establish a completer and more representative clinical situation of the patient. In addition, due to the complexity of this disease, one biomarker might not be enough for the diagnosis of NPSLE. To conclude, further studies are needed to better understand which is the role of these autoantibodies in the pathogenesis of NPSLE.

## Abbreviations

Acl: Anti-cardiolipin
AGA: Antibodies against gangliosides
AMPARs: AMPA-type glutamate receptors
ANAs: Anti-Nuclear Antibodies
ANFA: Autoantibodies targeting neurofilaments
Anti-ribP: Anti-ribosomal P protein antibodies
APCs: Antigen presenting cells
BAFF: B cell activating factor
BBB: Blood–brain barrier
BCR: B cell receptor
BCSFB: Blood-cerebrospinal fluid barrier
CNS: Central nervous system
DCs: Dendritic cells
DNase I: Deoxyribonuclease I
dsDNA: Double-strand DNA
EBNA-1: Epstein Barr virus nuclear antigen I
EBV: Epstein Barr virus
ENAs: Extractable nuclear antigens
FcγRs: Fcγ receptors
Gas6: Growth arrest‐specific 6
GCs: Germinal centers
GluR: Glutamate receptor
HLA: Human leukocyte antigen
ICAM-1: Intercellular adhesion molecule 1
ICs: Immune complexes
IFN-I: Type 1 interferons
IFN-α: Interferon-α
IFN-γ: Interferon-γ
IFN-γR: IFN-γ receptor
IgG: Immunoglobulin G
IL-1β: Interleukin 1β
IL-2: Interleukin-2
IL-21: Interleukin 21
IL-6: Interleukin 6
LAC: Lupus anticoagulant
MAP-2: Microtubule associated protein 2
NETs: Neutrophil extracellular trap
NMDA receptor: *N*-Methyl-d-aspartate receptor
NPSLE: Neuropsychiatric systemic lupus erythematosus
PECAM-1: Platelet endothelial cell adhesion molecule 1
PNS: Peripheral nervous system
PS: Phosphatidylserine
RNP: Ribonucleoprotein
SLE: Systemic lupus erythematosus
snRNPs: Small nuclear ribonucleoproteins
SS: Sjogren’s syndrome
T_FH_: CD4^+^ T follicular helper
T_h1_: CD4^+^ T helper 1
T_h17_: CD4^+^ T helper 17
Tim4: T cell immunoglobulin and mucin domain‐containing molecule 4
TLR: Toll-like receptor
TNF-α: Tumor necrosis factor
TNFR1: Tumor necrosis factor receptor 1
T_reg_: CD4^+^ T regulatory
VCAM-1: Vascular cell adhesion protein 1
